# MiR-193a-5p/ERBB2 act as concurrent chemoradiation therapy response indicator of esophageal squamous cell carcinoma

**DOI:** 10.18632/oncotarget.9444

**Published:** 2016-05-18

**Authors:** Cheng-Han Lin, Chen-Hsun Tsai, Ching-Tung Yeh, Jui-Lin Liang, Wan-Chun Hung, Forn-Chia Lin, Wei-Lun Chang, Hao-Yi Li, Yun-Chin Yao, Tai-I Hsu, Yu-Cheng Lee, Yi-Ching Wang, Bor-Shyang Sheu, Wu-Wei Lai, Marcus J. Calkins, Michael Hsiao, Pei-Jung Lu

**Affiliations:** ^1^ Institute of Clinical Medicine, College of Medicine, National Cheng Kung University, Tainan 704, Taiwan; ^2^ Institute of Basic Medical Sciences, College of Medicine, National Cheng Kung University, Tainan 704, Taiwan; ^3^ Department of General Surgery, Chi-Mei Medical Center, Liouying, Tainan 736, Taiwan; ^4^ Department of Radiation Oncology, National Cheng Kung University Hospital, Tainan 704, Taiwan; ^5^ Department of Internal Medicine, National Cheng Kung University Hospital, Tainan 704, Taiwan; ^6^ Clinical Medicine Research Center, National Cheng Kung University Hospital, Tainan 704, Taiwan; ^7^ Department of Pharmacology, College of Medicine, National Cheng Kung University, Tainan 704, Taiwan; ^8^ Department of Surgery Medicine, National Cheng Kung University Hospital, Tainan 704, Taiwan; ^9^ Genomics Research Center, Academia Sinica, Taipei 115, Taiwan

**Keywords:** miR-193a-5p, ERBB2, CCRT, esophageal squamous cell carcinoma, indicator

## Abstract

Concurrent chemoradiation therapy (CCRT) is the predominant treatment in esophageal cancer, however resistance to therapy and tumor recurrence are exceedingly common. Elevated ERBB2/Her2 may be at least partially responsible for both the high rates of recurrence and resistance to CCRT. This receptor tyrosine kinase is upregulated in 10–20% of esophageal squamous cell carcinoma (ESCC) tissues, and amplification of ERBB2 has been correlated with poor prognosis in esophageal cancer. Tissues from 131 ESCC patients, along with cell and animal models of the disease were used to probe the underlying mechanisms by which ERBB2 upregulation occurs and causes negative outcomes in ESCC. We found that overexpression of ERBB2 inhibited radiosensitivity *in vitro*. Furthermore, miR-193a-5p reduced ERBB2 expression by directly targeting the 3′UTR. Increased miR-193a-5p enhanced radiosensitivity and inhibited tumorigenesis *in vitro* and *in vivo*. Additionally, low miR-193a-5p expression correlated with poor prognosis in ESCC patients, and ESCC patients with good CCRT response exhibited higher miR-193a-5p expression. Our data suggest that patients with high miR-193a-5p will likely benefit from CCRT treatment alone, however a combination of CCRT with Herceptin may be beneficial for patients with low miR-193a-5p expression.

## INTRODUCTION

Esophageal cancer is the eighth most common cancer worldwide, and accounts for 3.2% of all carcinomas and 4.9% of all carcinoma-related deaths. Two main histological types comprise esophageal cancer: esophageal squamous cell carcinoma (ESCC) and esophageal adenocarcinoma (EAC), with ESCC being the predominant histological type in Asian countries. The risk factors of ESCC include smoking, alcohol use, betel nut consumption, and HPV infection [1, 2]. Overall, esophageal cancer patients face two- and five-year survival rates of only 50% and 20%, respectively [3]. Because of these high rates of occurrence and mortality, esophageal cancer requires more effective treatment strategies.

Currently, the National Comprehensive Cancer Network guidelines for esophageal and esophagogastric junction cancers indicate that surgery and concurrent chemoradiation therapy (CCRT) are the primary treatments for cancer of the esophagus. However, patients with late stage disease usually cannot undergo surgery due to cancer cell invasion into the aorta, vertebral body, or trachea. Previous studies have demonstrated better survival rates for esophageal cancer patients after receiving CCRT compared with radiation therapy alone [4, 5]. Nevertheless, a large portion of patients suffer from tumor recurrence, and more than 50% of tumor recurrences occur within one year after therapy [6]. Therefore, the identification of molecules involved in both tumorigenesis and regulation of the CCRT response may lead to improved therapies for esophageal cancer.

ERBB2 belongs to the HER family of transmembrane tyrosine kinase receptor proteins. Overexpression of HER family members is associated with poor outcomes in several tumor types. ERBB2 plays an important role in cell growth, survival, and differentiation in normal cells, and overexpression of ERBB2 promotes tumor growth, metastasis, and angiogenesis in cancer [7, 8]. In both EAC [9] and ESCC [10–12], ERBB2 overexpression has been reported. Hence, ERBB2 is considered a potential therapeutic target for esophageal cancer. However, the underlying molecular mechanism of how ERBB2 regulates ESCC tumorigenesis remains unclear.

MicroRNAs are small RNAs that are transcribed from noncoding sequence by RNA polymerase II [13], and regulate RNAs post-transcriptionally. Researchers have found that microRNA dysregulation is involved in many diseases, including cancer [14, 15]. In ESCC, microRNAs have been shown to regulate cell proliferation and are associated with patient survival [16, 17]. According to comparative microRNA expression profiles between normal and tumor tissues in ESCC, the expression of approximately 20 microRNAs is dysregulated [18–20]. However, the clinical significance of cancer-related microRNAs in esophageal cancer remains largely unclear. To better understand the role of microRNAs in clinical disease, we identified novel microRNAs associated with ESCC tumorigenesis and investigated the molecular mechanisms by which they act.

In ESCC, 10–20% of tumors show ERBB2 up-regulation, and amplification of ERBB2 is correlated with poor prognosis [21, 22]. We hypothesized that ERBB2 overexpression induces CCRT resistance, which causes the poor prognosis for ESCC patients. We then tested and studied the microRNA based regulation of ERRB2 in the CCRT response. In this study, we found that down-regulation of ERBB2 enhanced radiosensitivity. MiR-193a-5p regulated ERBB2 expression by directly targeting to the ERBB2-3′UTR, and was found to enhance radiosensitivity and inhibit tumorigenesis *in vitro* and *in vivo*. MiR-193a-5p expression level was inversely correlated with ERBB2, and low miR-193a-5p expression was correlated with poor prognosis in ESCC patients. We further found that CCRT resistant cells contained high levels of ERBB2 and low miR-193a-5p expression. Overexpression of miR-193a-5p enhanced CCRT response in KYSE cells. Furthermore, ESCC patients with good CCRT response had higher miR-193a-5p expression. Finally, overexpression of miR-193a-5p disrupted Herceptin dependent growth inhibition through ERBB2 down-regulation.

## RESULTS

### High ERBB2 expression enhances radiation resistance that relates to poor prognosis in ESCC

**Figure 1:**
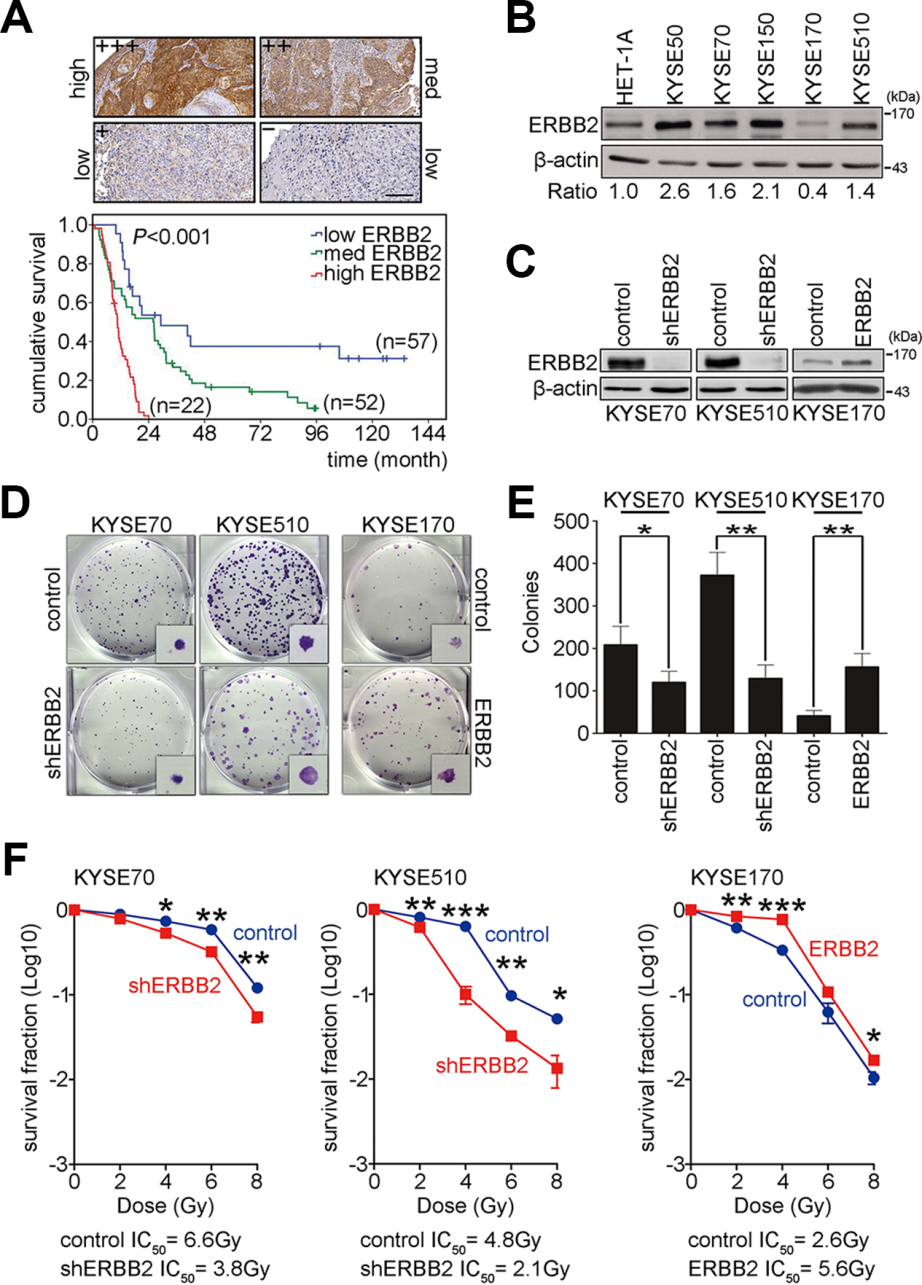
ERBB2 overexpression enhances tumorigenesis and radiation resistance to cause poor prognosis in ESCC (**A**) Immunohistochemistry was conducted to detect the expression of ERBB2 in ESCC tumor samples. Scale bar, 200 μm. Kaplan-Meier plots of overall survival in 131 ESCC patients stratified by ERBB2 expression level. (**B**) Western blotting showed endogenous ERBB2 expression in normal human esophageal epithelial cells and KYSE cells. (**C**) Western blotting showed the expression of ERBB2 in ERBB2-down-regulated stable cell lines (KYSE70 and KYSE510) and an ERBB2-up-regulated stable cell line (KYSE170). (**D** and **E**) The colony formation assay was used to analyze ERBB2-dependent tumorigenesis *in vitro*; *P < 0.05; **P < 0.01. (**F**) The survival fraction was used to represent radiosensitivity in KYSE cells; *P < 0.05; **P < 0.01; ***P < 0.001.

To evaluate whether ERBB2 is a good biomarker of prognosis for ESCC patients, immunohistochemistry was used to detect the expression of ERBB2 in 131 ESCC patients. The expression intensity of ERBB2 was scored and classified into 4 groups (+++, ++, +, –). Samples were then categorized as high ERBB2 expression (+++), medium ERBB2 expression (++), or low ERBB2 expression (+, –) (Figure 1A). The overall survival of these 131 ESCC patients was analyzed using the Kaplan-Meier method and groups were compared with a log-rank test. The results showed that high ERBB2 expression was correlated with poor prognosis for ESCC patients (Figure 1A). To characterize the function of ERBB2 in ESCC, HET-1A (normal human esophageal epithelial cells) and KYSE cells were used as a model system. Western blotting was used to detect the expression of ERBB2. The intensity of ERBB2 was normalized to β-actin (internal control), and the ratio of ERBB2 expression in KYSE cells to expression in normal HET-1A cells was calculated. In four of the five KYSE cell lines (excepting KYSE170) ERBB2 expression was at least 1.4-fold higher than normal cells (Figure 1B). We then generated two ERBB2 down-regulated stable cell lines in KYSE70 and KYSE510 cells and one ERBB2 up-regulated stable cell line in KYSE170 cells to study ERBB2-dependent tumorigenesis (Figure 1C). The AKT pathway is downstream of ERBB2, so we monitored levels of activated AKT in these cell lines. Western blotting showed that the expression of pAKT^308^ and pAKT^473^ were decreased in shERBB2_KYSE70 cells and increased in ERBB2_KYSE170 cells. These results indicated that modulation of ERBB2 levels in stable cell lines can produce the expected ERBB2-dependent signaling processes in KYSE cells (Supplementary Figure S1). A colony formation assay was used to verify tumorigenesis in KYSE cells. The results showed that tumorigenesis decreased 58% and 35% in shERBB2_KYSE70 and shERBB2_KYSE510 cells, respectively. Tumorigenesis increased 3.8-fold in ERBB2_KYSE170 cells (Figure 1D, 1E). These results indicated that ERBB2 expression promoted tumorigenesis in KYSE cells.

Radiotherapy is the common treatment for ESCC. Poor radiosensitivity leads to poor survival of the patients. To test whether up-regulated ERBB2 expression decreased radiosensitivity, the surviving fraction was calculated in ERBB2 down-regulated and up-regulated stable cell lines after irradiation. The IC_50_ radiation dose was calculated to represent radiosensitivity. The IC_50_ in ERBB2 down-regulated cells was significantly decreased when compared with vector control cells (shERBB2 IC_50_/control IC_50_: 3.8 Gy/6.6 Gy in KYSE70 cells; 2.1 Gy/4.8 Gy in KYSE510 cells; Figure 1F). The IC_50_ in ERBB2-overexpressing cells was significantly increased compared with vector control cells (ERBB2 IC_50_/control IC_50_: 5.6 Gy/2.6 Gy in KYSE170 cells; Figure 1F). The above results indicated that high ERBB2 is a potential prognosis marker in ESCC patients. ERBB2 promotes tumorigenesis, and the up-regulation of ERBB2 decreases radiosensitivity in KYSE cells.

### MiR-193a-5p inhibits ERBB2 expression and enhances radiosensitivity

**Figure 2:**
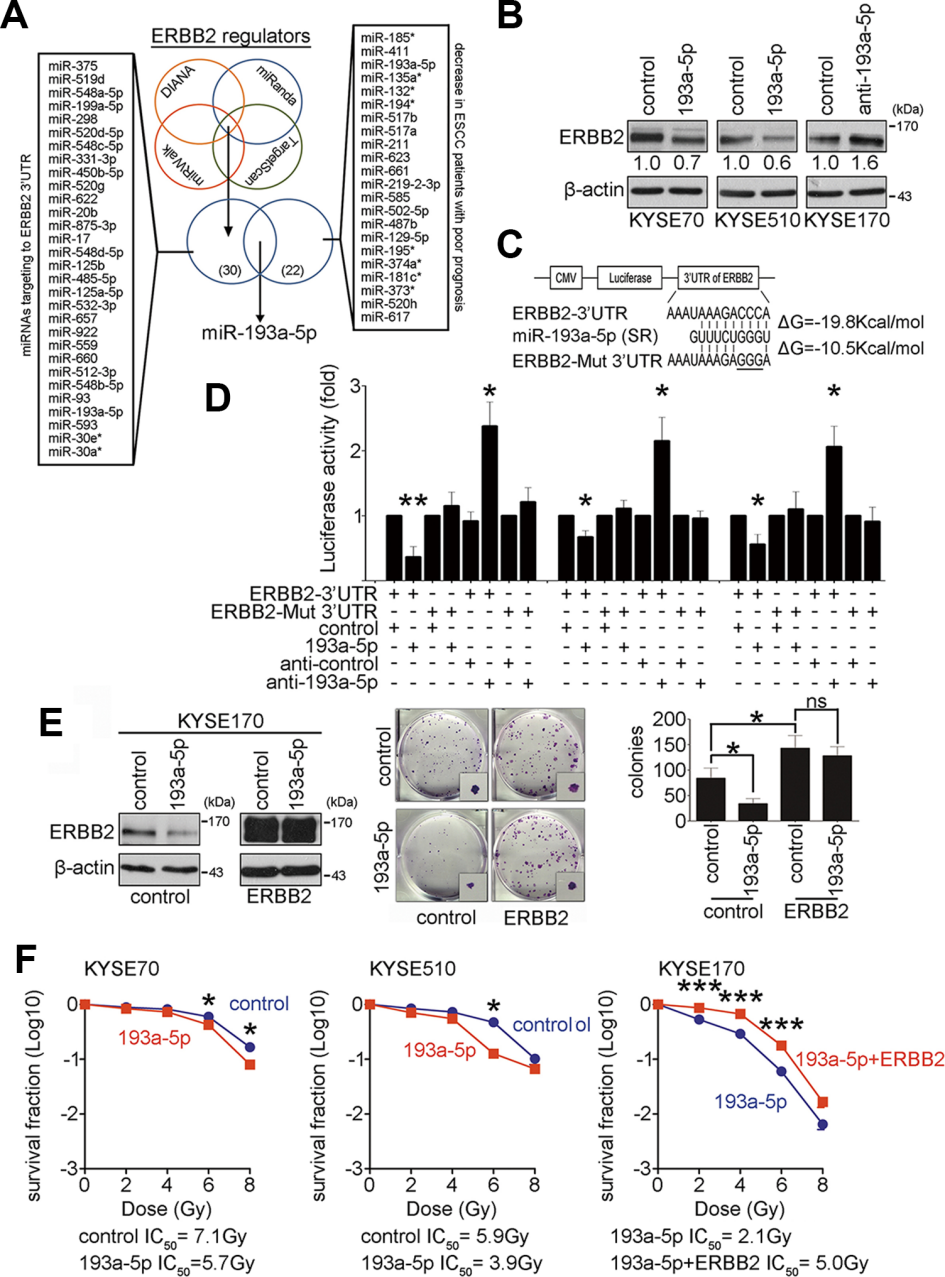
Down-regulation of ERBB2 is regulated by miR-193a-5p to enhance radiosensitivity (**A**) Bioinformatics websites and a human microarray data were employed to predict miRNAs that regulate ERBB2 expression. (**B**) Western blotting showed ERBB2 expression after manipulating miR-193a-5p in KYSE cells. (**C** and **D**) The luciferase reporter assay was used to demonstrate direct binding between the seed region (SR) of miR-193a-5p and ERBB2-3′UTR or ERBB2-Mut 3′UTR; *P < 0.05; **P < 0.01. (**E**) A colony formation assay was used to verify the involvement of miR-193a-5p in ERBB2-dependent tumorigenesis *in vitro*; *P < 0.05; ns, non-significant. (**F**) The survival fraction was used to represent radiosensitivity in KYSE cells; *P < 0.05; ***P < 0.001.

MicroRNAs are potential biomarkers in many diseases, including ESCC. To identify and characterize microRNAs that may regulate ERBB2 expression in ESCC, four bioinformatics websites were employed to predict microRNAs that target the ERBB2 3′UTR. In addition, a human microRNA microarray was used to identify microRNAs involved in ESCC tumorigenesis. Nine pairs of esophageal tumor and adjacent normal epithelial tissues with good prognosis (survival period > 25 month) or poor prognosis (survival period < 18 month) were used to evaluate the differential microRNA expression profiles. Twenty-two tumor-suppressive microRNAs, which were decreased in ESCC patients with poor prognosis, were selected from the array results. After comparing the predicted microRNAs from databases and selected microRNAs from the microarray, miR-193a-5p was chosen for further examination (Figure 2A and Supplementary Figure S2). To verify whether ERBB2 expression was suppressed by miR-193a-5p in KYSE cells, miR-193a-5p precursors and inhibitors were transiently transfected into KYSE cells. Western blotting showed ERBB2 expression after transfection. MiR-193a-5p overexpression caused ERBB2 to decrease to 70% and 60% of control levels in KYSE70 and KYSE510 cells, respectively. The down-regulation of miR-193a-5p increased ERBB2 expression by 1.6-fold in KYSE170 cells (Figure 2B). The target genes of miR-193a-5p were also predicted by bioinformatics websites. ERBB2, YES1, and KDELR3 expression were examined by western blotting after miR-193a-5p manipulation in KYSE cells. The results showed that ERBB2 was the only target protein repressed by miR-193a-5p (Figure 2B and Supplementary Figure S3). To study whether miR-193a-5p suppressed ERBB2 expression by directly binding to the ERBB2 3′UTR, a luciferase reporter assay was used in KYSE cells. According to a sequence alignment, the seed region of human miR-193a-5p formed a complimentary match with the ERBB2 3′UTR target sequence in different mammals (Supplementary Figure S4). We then generated two luciferase reporter constructs. One construct linked luciferase with the wild type ERBB2 3′UTR target sequence (ERBB2-3′UTR), and the other construct contained a mutation in the ERBB2 3′UTR target sequence (CCC to GGG, ERBB2-Mut-3′UTR; Figure 2C). The results of the reporter assay showed that miR-193a-5p overexpression reduced luciferase activity approximately 35 to 60%, whereas miR-193a-5p down-regulation increased luciferase activity by 2.3-fold in the ERBB2-3′UTR group. In the ERBB2-Mut-3′UTR group, luciferase activity was unaffected by miR-193a-5p manipulation (Figure 2D). These results suggest that miR-193a-5p represses ERBB2 expression by directly binding to the ERBB2 3′UTR. Since ERBB2 promotes tumorigenesis and decreases radiosensitivity in KYSE cells (Figure 1), we sought to determine whether ERBB2-dependent tumorigenesis and radiosensitivity are regulated by miR-193a-5p. ERBB2-overexpressing stable cells were generated in KYSE170 cells, and miR-193a-5p precursors were transiently transfected in these cells. The colony formation assay and IC_50_ radiation dose were used to verify the effects on tumorigenesis and radiosensitivity, respectively. Figure 2E showed that in the control group, miR-193a-5p overexpression decreased ERBB2 expression and inhibited tumorigenesis *in vitro*. In ERBB2-overexpressing cells, the regulatory ability of miR-193a-5p was abolished because the ERBB2-overexpressing plasmid did not include the ERBB2 3′UTR (Figure 2E). These results demonstrated that ERBB2-dependent tumorigenesis is regulated by miR-193a-5p. Transient expression of miR-193a-5p successfully decreased ERBB2 expression in KYSE70 and KYSE510 cells (Figure 2B). The IC_50_ in miR-193a-5p-overexpressing cells was significantly decreased compared with the vector control group (193a-5p IC_50_/control IC_50_: 5.7 Gy/7.1 Gy in KYSE70 cells; 3.9 Gy/5.9 Gy in KYSE510 cells; Figure 2F). In KYSE170 cells, the IC_50_ in ERBB2 and miR-193a-5p co-expressing cells was significantly increased compared with miR-193a-5p-expressing cells (193a-5p+ERBB2 IC_50_/193a-5p IC_50_: 5.0 Gy/2.1 Gy; Figure 2F). The CCRT response was further investigated in KYSE170 cells. The cisplatin IC_50_ in miR-193a-5p-overexpressing cells was decreased compared with vector control group, and the cisplatin IC_50_ in ERBB2 and miR-193a-5p co-expressing cells was increased compared with miR-193a-5p-overexpressing or vector control group (control IC_50_/193a-5p IC_50_/193a-5p + ERBB2 IC_50_: 27.1 μM/12.0 μM/42.3 μM; Supplementary Figure S6). These results suggested that miR-193a-5p-mediated ERBB2 down-regulation inhibits tumorigenesis *in vitro* and increases CCRT sensitivity in KYSE cells.

### MiR-193a-5p inhibits tumor growth by down-regulating ERBB2 *in vitro* and *in vivo*

**Figure 3:**
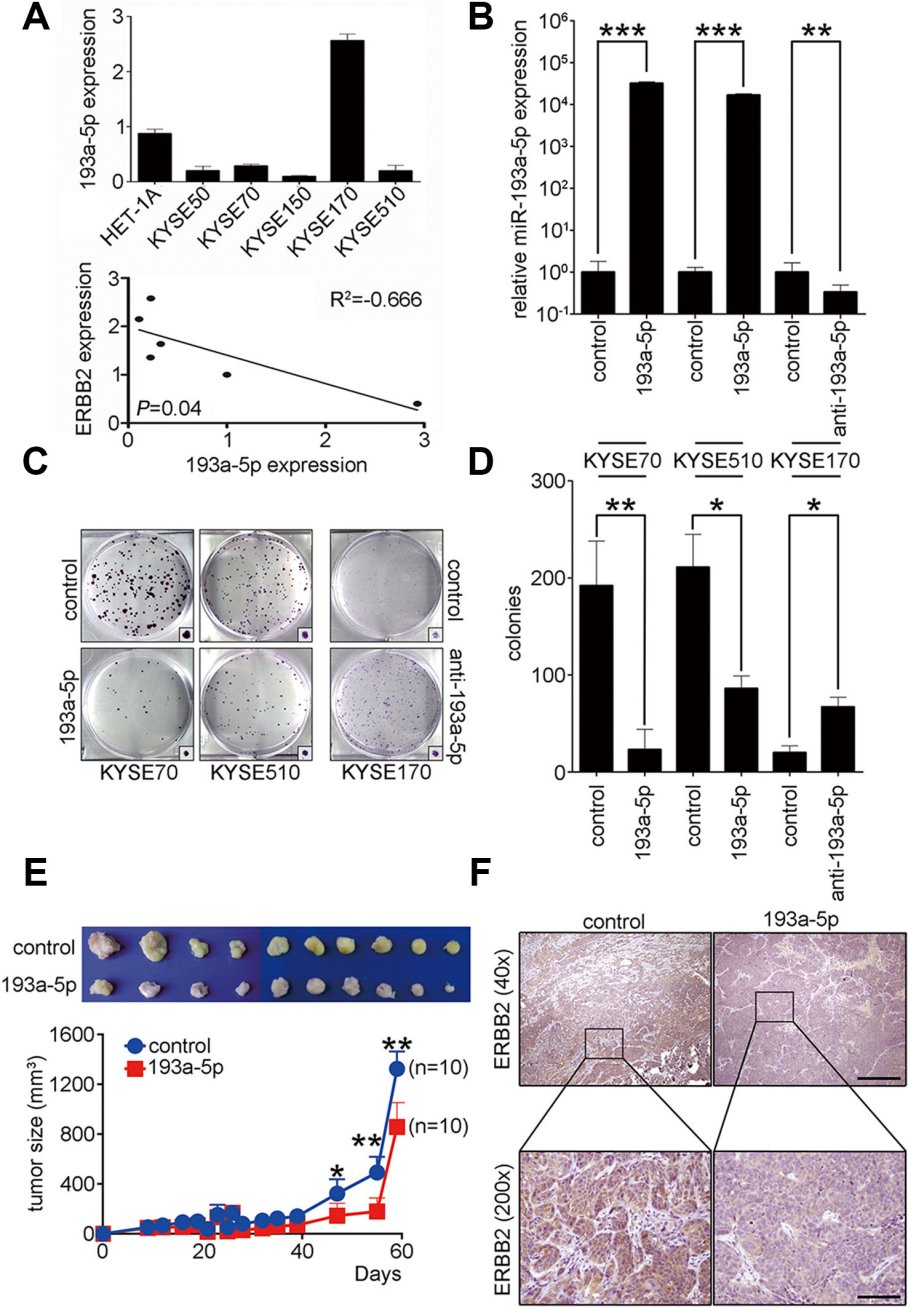
Up-regulation of miR-193a-5p decreases ERBB2 expression level to suppress ESCC tumorigenesis *in vitro* and *in vivo* (**A**) Real-time PCR was used to evaluate miR-193a-5p expression in HET-1A cells and KYSE cells. ERBB2 was inversely correlated with miR-193a-5p expression in KYSE cells; R^2^ = – 0.666; *P* = 0.04. (**B**) A miR-193a-5p precursor was transfected into KYSE70 and KYSE510 cells; ***P < 0.001. MiR-193a-5p inhibitors were transfected into KYSE170 cells; **P < 0.01. (**C** and **D**) The effect of miR-193a-5p on tumorigenesis *in vitro* was analyzed using a colony formation assay in KYSE cells; *P < 0.05; **P < 0.01. (**E**) A xenograft mouse model was used to analyze the effect of miR-193a-5p on tumorigenesis *in vivo*; *P < 0.05. (**F**) Immunohistochemistry was used to verify the expression of ERBB2 in xenograft tumors. Scale bar, 500 μm (40×); 50 μm (200×).

To investigate the role of miR-193a-5p in KYSE cells, real-time PCR was used to examine endogenous miR-193a-5p expression in HET-1A and KYSE cells. The results showed lower miR-193a-5p expression in KYSE50, KYSE70, KYSE150, and KYSE510 cells compared to HET-1A cells. MiR-193a-5p expression was higher in KYSE170 than HET-1A cells (Figure 3A). Combining the results from Figure 1B and Figure 3A, we found that expression of ERBB2 was inversely correlated with miR-193a-5p in KYSE cells. To study the role of miR-193a-5p in ESCC tumorigenesis, miR-193a-5p precursors and inhibitors were transiently transfected into KYSE cells. Real-time PCR results showed that miR-193a-5p expression was successfully manipulated after transfection (Figure 3B). A colony formation assay and soft agar assay were used to investigate tumorigenesis *in vitro* after miR-193a-5p manipulation in KYSE cells (Figure 3C–3D and Supplementary Figure S5). The results showed that miR-193a-5p overexpression reduced the colony formation ability by approximately 88% and 41% in KYSE70 and KYSE510 cells, respectively. The down-regulation of miR-193a-5p increased colony formation ability by 3-fold in KYSE170 cells (Figure 3D). The soft agar assay represents anchorage-independent growth in *in vitro* tumorigenesis and showed a 62% and 47% decrease in miR-193a-5p-overexpressing KYSE70 and KYSE510 cells, respectively. The down-regulation of miR-193a-5p increased anchorage independent growth by 2-fold in KYSE170 cells (Supplementary Figure S5). To further study the function of miR-193a-5p *in vivo*, a miR-193a-5p-overexpressing stable cell line in KYSE70 cells was generated and subcutaneously injected into immunodeficient mice. The tumor sizes were measured weekly. The mice were sacrificed, and the tumors were collected two months after tumor cells injection. Tumors with miR-193a-5p overexpression were significantly smaller than the control groups (Figure 3E). The expression of ERBB2 in xenograft tumors was verified by immunohistochemistry. Indeed, the expression of ERBB2 was significantly decreased in miR-193a-5p-overexpressing tumors (Figure 3F). These results indicated that miR-193a-5p inhibited tumor growth by decreasing ERBB2 expression *in vitro* and *in vivo*.

### MiR-193a-5p is a novel prognostic marker for ESCC

**Figure 4:**
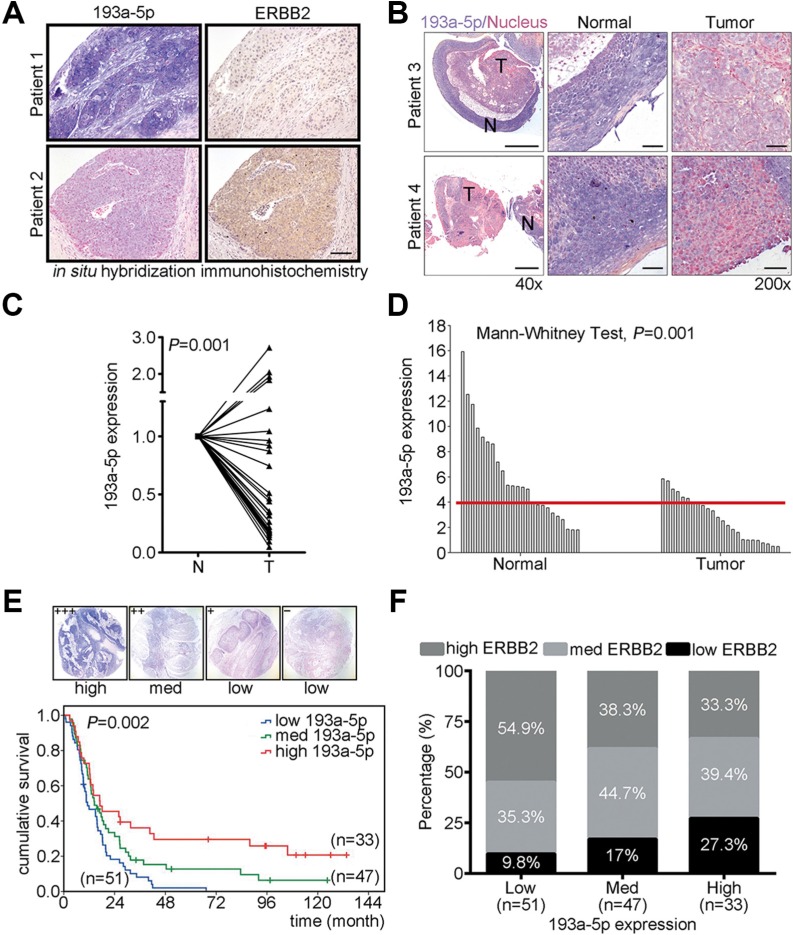
Down-regulation of miR-193a-5p is inversely correlated with ERBB2 expression level and is correlated with poor prognosis in ESCC (**A**) Immunohistochemistry and *in situ* hybridization were conducted to detect the expression of ERBB2 and miR-193a-5p in ESCC tumor samples. Scale bar, 100 μm. (**B**) *In situ* hybridization was conducted to examine the expression of miR-193a-5p in ESCC specimens. Scale bar, 500 μm (40×) and 50 μm (200×). (**C**) Real-time PCR showed the expression of miR-193a-5p in N-T paired specimens of twenty-eight patients with esophageal cancer; *P* = 0.001. (**D**) In unpaired normal and tumor tissues, miR-193a-5p was detected and analyzed using a Mann-Whitney test; *P* = 0.001. The cutoff value was 3.91, as determined by a ROC curve. (**E**) Kaplan-Meier plots of the overall survival of 131 ESCC patients stratified by miR-193a-5p expression level. (**F**) Spearman's correlation analysis showed the inversely correlation between ERBB2 and miR-193a-5p; *P* = 0.015, correlation coefficient = – 0.213.

To verify the correlation of ERBB2 and miR-193a-5p in ESCC patients, immunohistochemistry and *in situ* hybridization were performed to examine both ERBB2 and miR-193a-5p expression in clinical tissues (Figure 4A). In patients with high miR-193a-5p, the expression of ERBB2 was lower compared with patients with low miR-193a-5p (Figure 4A). To verify whether miR-193a-5p is a potential prognostic marker in ESCC, *in situ* hybridization and real-time PCR were used to examine miR-193a-5p expression in tumor tissues from 28 patients. *In situ* hybridization showed that miR-193a-5p was decreased in tumor tissue compared with adjacent normal tissue (purple color indicates miR-193a-5p and red color indicates nucleus, Figure 4B). In NT paired analysis, the tissues were collected from the same patient, or non-NT paired analysis, all collected tissues form patients, miR-193a-5p was significantly decreased in tumor tissue (Figure 4C–4D). To evaluate whether miR-193a-5p was a good prognostic marker in ESCC patients, *in situ* hybridization was used to detect the expression of miR-193a-5p in 131 ESCC patients. The expression intensity of miR-193a-5p was scored and classified into 4 groups (+++, ++, +, –). High miR-193a-5p expression (+++), medium miR-193a-5p expression (++), and low miR-193a-5p expression (+, –) were further sub-divided from these groups (Figure 4E). The overall survival of these 131 ESCC patients was analyzed using the Kaplan-Meier method and groups were compared with a log-rank test. The results showed that low miR-193a-5p expression was correlated with poor prognosis for ESCC patients (Figure 4E). To further investigate the correlation between miR-193a-5p and ERBB2, the percentage of high/med/low ERBB2 was analyzed in low/med/high miR-193a5p groups. In low miR-193a-5p group, 54.9% were high ERBB2, 35.3% were medium ERBB2, and 9.8% were in the low ERBB2 group. In the medium miR-193a-5p group, 38.3% showed high ERBB2, 44.7% exhibited medium ERBB2, and 17% were in the low ERBB2 group. In the high miR-193a-5p group, 27.3% were high ERBB2, 39.4% were medium ERBB2, and 33.3% were low ERBB2 (Figure 4F). The Spearman's correlation analysis was used to investigate the expression of ERBB2 in ESCC patients within different miR-193a-5p expression groups. The results showed that ERBB2 expression was inversely correlated with miR-193a-5p in these groups (correlation coefficient = −0.213, *P* = 0.015). These results suggested that miR-193a-5p is a potential diagnostic and prognostic marker in ESCC.

### High miR-193a-5p expression is correlated with good CCRT response in ESCC

**Figure 5:**
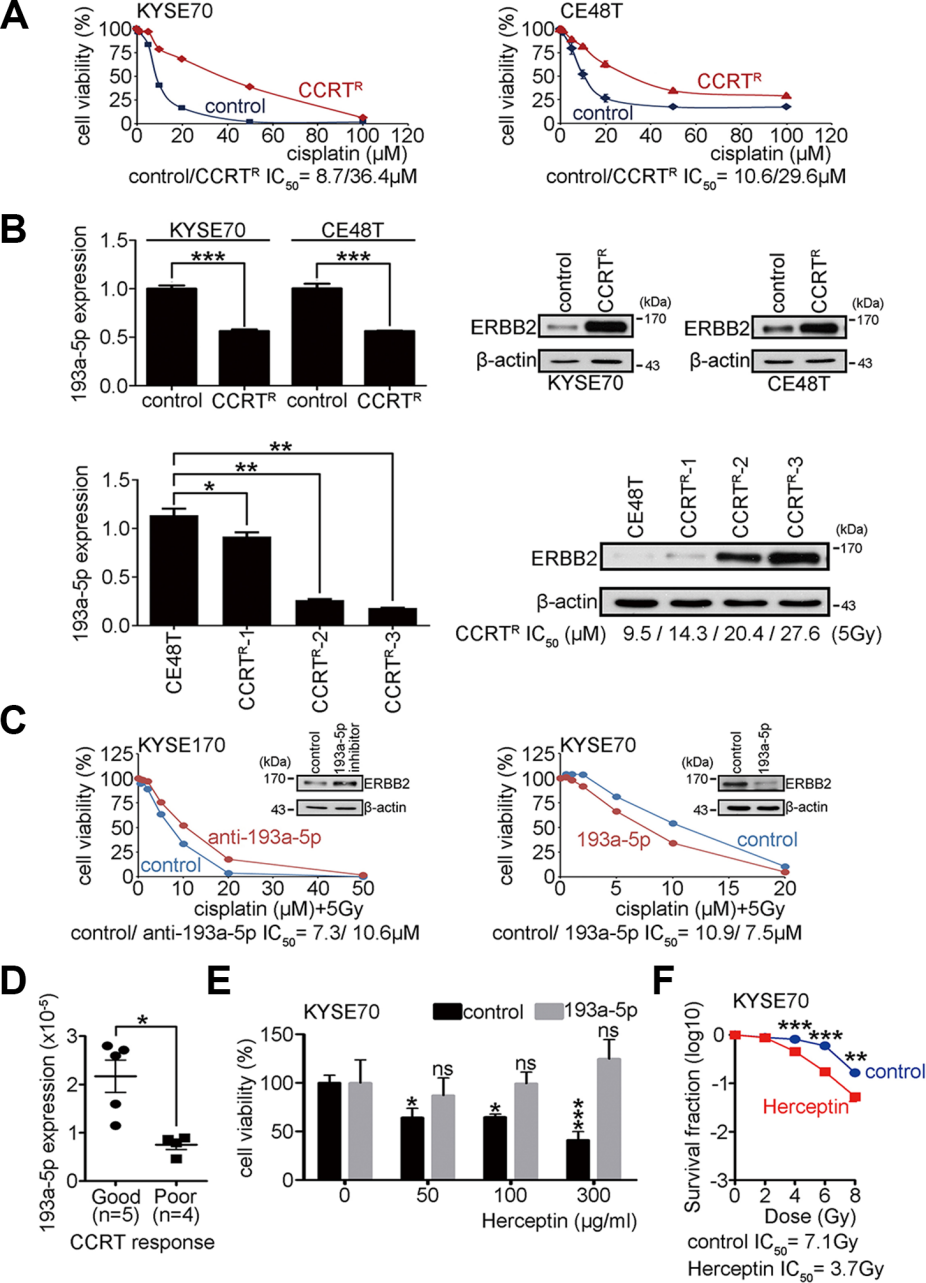
Overexpression of miR-193a-5p decreases ERBB2 expression and enhances the CCRT response in ESCC (**A**) CCRT^R^ KYSE70 and CE48T cells were generated, and cell viability was verified using the MTT assay after treatment with different dosages of cisplatin combined with 5 Gy of irradiation. (**B**) Real-time PCR showed the expression of miR-193a-5p in CCRT^R^ cells, and Western blotting showed ERBB2 expression in CCRT^R^ cells; *P < 0.05; **P < 0.01. (**C**) The MTT assay was used to verify cell viability after CCRT treatment. (**D**) Real-time PCR showed the expression of miR-193a-5p in ESCC patients with a good or poor CCRT response. (**E**) The MTT assay was used to verify cell viability after Herceptin treatment in KYSE70 cells; *P < 0.05; ***P < 0.001. (**F**) The survival fraction was used to represent radiosensitivity after Herceptin treatment in KYSE70 cells; **P < 0.01; ***P < 0.001.

Surgery and concurrent chemoirradiation treatment (CCRT) are the primary treatments for esophageal carcinoma. Investigating molecules that regulate the CCRT response may lead to new therapies that will help to improve ESCC patient survival. To study whether the miR-193a-5p expression level influences the CCRT response in ESCC, CCRT-resistant cell lines were previously generated from KYSE70 and esophageal epidermoid carcinoma (CE48T) cells [23]. The resistant cells were treated with various concentrations of cisplatin combined with 5 Gy of irradiation to evaluate the CCRT response. The MTT assay was used to analyze cell viability. The IC_50_ cisplatin concentration was calculated to represent the CCRT response under 5 Gy of irradiation. A higher IC_50_ indicated more resistance to CCRT and was considered a poor CCRT response. The results showed that the IC_50_ in CCRT-resistant cells was significantly increased when compared with control cells (CCRT^R^ IC_50_/control IC_50_: 36.4 μM/8.7 μM in KYSE70 cells; CCRT^R^ IC_50_/control IC_50_: 29.6 μM / 10.6 μM in CE48T cells; Figure 5A). Real-time PCR was used to measure miR-193a-5p expression in CCRT-resistant cells. The results showed an approximately 50% reduction in miR-193a-5p expression in CCRT-resistant cells compared to parental control cells (Figure 5B). The expression of ERBB2 was also examined by western blotting in control and CCRT-resistant cells. The results showed that ERBB2 was significantly increased in CCRT-resistant cells (Figure 5B). To examine whether the up-regulation of ERBB2 and down-regulation of miR-193a-5p were a selective or adaptive response after CCRT treatment, CE48T cells were exposed to several cycles of CCRT treatment (CCRT^R^-1, CCRT^R^-2, and CCRT^R^-3). The expression of ERBB2 and miR-193a-5p was evaluated by western blotting and real-time PCR, respectively. Using 5 Gy of irradiation, the IC_50_ of cisplatin was 9.5 μM in parental CE48T cells, 14.3 μM in CCRT^R^-1 group, 20.4 μM in CCRT^R^-2 group, and 27.6 μM in CCRT^R^-3 group (Figure 5B). The results showed that the up-regulation of ERBB2 occurred in an irradiation dosage-dependent manner, and miR-193a-5p expression was inversely correlated with ERBB2 (Figure 5B). To characterize the functional role of miR-193a-5p in the CCRT response, KYSE cells treated with miR-193a-5p inhibitors and miR-193a-5p-overexpressing stable cells were treated with CCRT. The results showed higher cisplatin resistance in KYSE170 cells treated with miR-193a-5p inhibitors than control cells (Figure 5C). The cisplatin resistance was lower in stable miR-193a-5p-overexpressing KYSE70 cells than control cells (Figure 5C). Two independent miR-193a-5p-overexpressing KYSE70 cells were further used to investigate whether miR-193a-5p enhanced CCRT response in a dose dependent manner. The cisplatin resistance was lower in high miR-193a-5p-overexpressing than low miR-193a-5p-overexpressing KYSE70 cells (Supplementary Figure S7). The results indicated that high miR-193a-5p was correlated with a good CCRT response in KYSE170 and KYSE70 cells. ESCC patients who received CCRT treatment were grouped into good or poor CCRT responses according to the criteria described in a previous study [24, 25]. To verify whether miR-193a-5p is a potential biomarker for the CCRT response, five CCRT good response and four CCRT poor response patient RNA samples were used to evaluate miR-193a-5p expression by real-time PCR. The results showed that miR-193a-5p was significantly higher in the CCRT good response than poor response patients (Figure 5D).

Since ERBB2 was highly expressed in CCRT^R^ cells (Figure 5B), we utilized these cells to investigate whether miR-193a-5p enhance CCRT response through ERBB2 dependent pathway. Multiple concentrations of Herceptin (0, 50, 100, 300 μg/ml), a neutralized antibody of ERBB2, were used to treat KYSE70 cells. After 72 hour treatment, MTT assay was used to measure cell viability. In the control group, the cell viability was significantly decreased (0 μg/ml: 100 ± 17.5% / 50 μg/ml: 64.1 ± 21. 8% / 100 μg/ml: 64.7 ± 7.0% / 300 μg/ml: 41.1 ± 19.5%; Two-way ANOVA), however, in miR-193a-5p group, the growth inhibition of Herceptin was eliminated (0 μg/ml: 100 ± 21.6% / 50 μg/ml: 87.1 ± 16.5% / 100 μg/ml: 99.3 ± 10.8% / 300 μg/ml: 124.7 ± 18.1%; Figure 5E). To test whether Herceptin treatment increased radiosensitivity, the surviving fraction was calculated in KYSE70 cells treated with 300 μg/ml Herception before irradiation. The IC_50_ in Herceptin-treated cells was significantly decreased compared with the control group (Herceptin IC_50_/control IC_50_: 3.7 Gy/ 7.1 Gy; Figure 5F). We then further investigated whether Herceptin treatment increased cisplatin sensitivity in CCRT resistant cells, CE48T CCRT^R^ cells were treated with different concentrations of Herceptin (0, 10, and 100 μg/ml) and cisplatin sensitivity was investigated using the MTT assay. The IC_50_ of cisplatin in Herceptin treated CCRT resistant cells was significantly decreased compared with the control group (Herceptin 100 μg/ml IC_50_/10 μg/ml IC_50_/control IC_50_: 4.6 μM / 7.1 μM / 23.3 μM; Supplementary Figure S8). A xenograft mouse model was also used to investigate whether Herceptin combined CCRT treatment suppressed tumor growth *in vivo*. Parental or miR-193a-5p overexpressing KYSE70 cells were subcutaneously injected into immunodeficient mice. One week after tumor cell injection, mice were subdivided into (i) control, (ii) CCRT, and (iii) Herceptin combined with CCRT groups. Cisplatin treatment (2 mg/Kg) and irradiation (4Gy) were conducted once. Herceptin (4 mg/Kg) was intravenously injected to mice, twice a week. Mice received Herceptin treatment for one month. Tumor sizes were measured weekly from one week to six weeks after treatment. The results showed that Herceptin combined with CCRT treatment significantly suppressed tumor growth compared with CCRT alone or control groups. Herceptin did not enhance CCRT response in mice injected with miR-193a-5p-ovexpresing KYSE70 cells (Supplementary Figure S9). These results suggest that high expression of miR-193a-5p decreases the ERBB2 expression level, and thereby enhances the CCRT response in ESCC patients. Therefore, Herceptin combined with CCRT treatment may be a potential therapeutic strategy for ESCC patients with low miR-193a-5p expression.

## DISCUSSION

In 2012, esophageal cancer was the 8th leading cause of cancer death, and exhibits a low survival rate due to poor response to treatments and frequent tumor recurrence. Chemotherapy, radiotherapy, and CCRT are the most common treatments for patients with esophageal cancer. Therefore, the identification of molecules that can enhance radiosensitivity or inhibit drug resistance is urgently needed. In this study, we found that down-regulation of ERBB2 enhanced radiosensitivity of ESCC cell lines. Furthermore, miR-193a-5p, a miRNA that regulates ERBB2 expression by directly targeting to ERBB2-3′UTR, enhanced radiosensitivity and inhibited tumorigenesis *in vitro* and *in vivo*. MiR-193a-5p expression level was inversely correlated with ERBB2 and low miR-193a-5p expression was correlated with poor prognosis in ESCC patients. We further found that CCRT resistant cells exhibited high ERBB2 and low miR-193a-5p expression. Moreover, overexpression of miR-193a-5p enhanced CCRT response in KYSE cells. These results were validated in patient samples, by showing that ESCC patients with good CCRT response have higher miR-193a-5p expression. Overexpression of miR-193a-5p disrupted Herceptin dependent growth inhibition through ERBB2 down-regulation. According to our results, ERBB2 and miR-193a-5p were potential prognostic and CCRT response biomarkers in ESCC. Our data suggest that patients with high miR-193a-5p will likely benefit from CCRT treatment alone. A combination of CCRT with Herceptin may be recommended for patients with low miR-193a-5p.

The ERBB2 pathway is overexpressed in several cancers and contributes to cell growth and survival. In our study, we showed that ERBB2 was highly expressed in many ESCC tissues and high expression was associated with poor prognosis (Figure 1). Hence, down-regulation of this pathway may be a valuable therapeutic strategy for ESCC. Additionally, we found that miR-193a-5p regulates ERBB2 expression by directly targeting the 3′UTR of ERBB2 (Figure 2). MiR-193a-5p and ERBB2 expression were inversely correlated in clinical specimens (Figure 4). Similar findings were previously reported in both breast cancer and EAC [26, 27]. Because reduction of ERBB2 promoted tumor apoptosis in gastrointestinal adenocarcinomas [28], we hypothesized that miR-193a-5p may also induce tumor apoptosis. Hence, we examined apoptosis rates after overexpressing miR-193a-5p in an ESCC cell line. Western blotting showed an increase in cleaved PARP, and flow cytometry revealed a 6-fold increase in the sub-G1 population in miR-193a-5p-overexpressing KYSE70 cells (Supplementary Figure S10). Together, these findings suggest that regulation of the miR-193a-5p/ERBB2 pathway might be a valuable therapeutic target in ESCC.

The associations between microRNAs and cancers have recently been extensively studied; however, the application of microRNAs in cancer treatment remains in an early phase. Up to now, most research has focused on identifying and characterizing the roles of microRNAs in different cancers. For example, Hussey and colleagues used microarray to detect the differential miRNAs expression signatures in chemo-resistant esophageal cancer cell lines (both EAC and ESCC cells). After evaluation by Q-PCR, miR-455-3p, miR-200b-3p, let-7e-5p, miR-181b-5p, miR-125a-5p, miR-181a-5p, miR-200b-5p, miR-31-5p, and miR-200a-3p were significantly down-regulated in cisplatin resistant EAC cells. Conversely, miR-638 and miR-191-5p were found to be up-regulated in cisplatin resistant EAC cells. In cisplatin resistant ESCC cells, miR-130a-3p was increased, and in 5-FU (5-fluorouracil) resistant esophageal cancer cells, miR-378a-3p, miR-192-5p, miR-210-3p, miR-194-5p, miR-17-3p, miR-935, miR-125a-5p, miR-1226-3p, miR-99b-5p, and miR-18a-3p were significantly down-regulated, while miR-222-3p, miR193b-3p, miR-31-5p, miR-27b-3p, and miR-550a-3p were increased. The results from this extensive study clearly demonstrate that esophageal cancer cells have distinct miRNA expression profiles that differentially affect response to individual chemotherapeutic agents [29]. We found that miR193a-5p down-regulation was significantly associated with poor prognosis in ESCC patients (Figure 4). Recently, reduced expression of miR-193a-5p was shown to be involved in tumorigenesis and metastasis in non-small cell lung cancer, endometrioid endometrial carcinoma, and follicular lymphoma [30, 31]. Combined with our findings, these reports indicate that miR-193a-5p functions as a tumor suppressor in a variety of cancers.

MiR-193a-5p overexpression, or ERBB2 disruption, significantly enhanced radiosensitivity of KYSE cells (Figure 1F and Figure 2F). Therefore, the data suggest that miR-193a-5p/ERBB2 pathway might be an important target for treating ESCC patients who are resistant to chemotherapy, radiotherapy and/or CCRT treatment. In our results, CCRT-resistant cells exhibited low miR-193a-5p expression and high ERBB2 expression. Further, miR-193a-5p overexpression diminished ERBB2 levels and was related to a good CCRT response in KYSE cells. Our clinical specimens further confirmed this correlation (Figure 5D). Herceptin is a neutralized antibody for ERBB2 that blocks signalling through this pathway. One small phase II study has reported preliminary findings that evaluate Herceptin combined with cisplatin or docetaxel in Her2 positive gastric and/or GEJ adenocarcinoma patients. However, the results have not been published. Another phase II trial showed results from twenty-one patients with Her2 positive advanced gastric or GEJ adenocarcinoma and treated with cisplatin and Herceptin. The total response rate was only 30%. Importantly, both miR-193a-5p and Herceptin treatments were effective at reducing either the cisplatin concentration or radiation dosage necessary to induce cell death. Our findings suggest that Herceptin combined with radiation therapy may eliminate the side effects of cisplatin, and provide a novel therapeutic method for treating ESCC patients. In addition, our findings suggest that miR-193a-5p might be a valuable predictive biomarker of the CCRT response in ESCC patients.

## MATERIALS AND METHODS

### Clinical specimens

Primary esophageal tumors and adjacent matched normal esophageal tissue were obtained from the National Cheng Kung University Hospital (Tainan, Taiwan). This study received *Institutional Review Board* approval (IRB number: A-ER-102-228). The carcinoma samples were obtained from a resection of the esophageal tumors, which were histologically examined for the presence of tumor tissue in hematoxylin and eosin-stained sections.

### Cell culture and reagents

The ESCC cell lines were cultured in RPMI-1640 medium containing 10% fetal bovine serum (FBS; Hyclone, Logan, YT, USA) and 100 units/ml penicillin/streptomycin (Caisson, North Logan, UT, USA). HET-1A human esophageal epithelial cell lines were cultured in bronchial epithelial cell growth medium (BEGM). All cells were maintained at 37°C in an atmosphere containing 5% CO_2_. The artificial miRNA precursors were purchased from Applied Biosystems (Applied Biosystems, Branchburg, NJ, USA). Inhibitor molecules were purchased from Exiqon (Exiqon A/S, Denmark). Control and ERBB2 shRNA constructs were purchased from the National RNAi Core Facility Platform (Taiwan).

### Quantitative real-time PCR (Q-PCR)

Total RNA isolation and reverse transcription were conducted from cells. The amplification and detection of specific products were performed with an ABI detection system with the cycle profile according to the TaqMan qRT–PCR miRNA Detection Kit (Applied Biosystems, Branchburg, NJ, USA). Relative gene expression was calculated by comparing the cycle thresholds for each target PCR. The target PCR Ct values were normalized by subtracting the internal control RNU6B snoRNA Ct value.

### MTT assay

Cells were plated onto 96-well plates and cultured overnight for complete cell attachment. The medium was removed and replaced with 0.33 mg/ml 3-(4,5-dimethylthiazol-2-yl)-2,5-diphenyl-5H-tetrazolium bromide (MTT, Sigma, USA) in RPMI for 2 hr. After incubation, the absorbance at 570 nm was determined using an ELISA reader. To verify the effect of the CCRT response, cells were treated with different dosages of cisplatin combined with 5 Gy irradiation.

### Colony formation, soft agar, and foci formation assays

The colony formation, soft agar, and foci formation assays were performed on a six-well plate. For colony formation, the cells were seeded, and the plates were incubated at 37°C in 5% CO_2_. After 8–16 days, the plates were stained with 1% crystal violet. The colony number in each well was counted. A single colony was defined to consist of at least 30 cells. In the soft agar assay, cells were suspended in 0.35% agarose gel contained RPMI medium with 10% FBS. Two weeks later, the plates were stained with MTT, and the colony number was counted. In the foci formation assay, cells were seeded onto six-well plates for six hours, and were irradiated with the indicate dose of radiation using a six megavoltage photon. Two weeks later, the plates were stained with 1% crystal violet. The colony number in each well was counted. The survival fraction was obtained by dividing the colony number of the cells treated with the indicated radiation dose by the colony number of control cells.

### Xenograft tumor growth

NOD-SCID mice were obtained from the National Cheng Kung University Laboratory Animal Center. All mice were maintained under standard protocols, and the experiment was approved by the Institutional Animal Care and Use Committee, National Cheng Kung University (IACUC Approval No: 101026). For xenograft tumor growth, each mouse was subcutaneously inoculated with 1 × 10^6^ KYSE70 cells mixed with Matrigel (BD Biosciences) (1:1) in total of 100 μl. Tumor sizes were measured weekly.

### Western blotting

For immunoblotting analysis, protein lysates were loaded onto a 10% SDS-polyacrylamide gel for electrophoresis and transferred to a PVDF membrane. Proteins were identified by incubating the membrane with primary antibodies followed by horseradish peroxidase-conjugated secondary antibodies and enhanced chemiluminescence solution (NEN Life Science, Boston, MA, USA). The primary antibodies used in this study were AKT, pAKT308, and pAKT473 from Cell Signaling Technology and ERBB2, YES1, and KDELR3 from Abcam.

### *In situ* hybridization

Tissue sections were dewaxed and rehydrated. Next, 4% paraformaldehyde was used to fix the sections, and the sections were treated with acetylation solution. Sections were then blocked with pre-hybridization solution and incubated with digoxigenin (Dig)-conjugated hsa-miR-193a-5p probes (Exiqon) or Dig-conjugated control probes with a scrambled sequence (Exiqon) overnight at 50°C. After extensive washing with SCC buffer, the sections were incubated with an AP-conjugated anti-digoxigenin antibody (Biochain) overnight at 4°C. After washing twice with alkaline phosphatase buffer, the sections were reacted with NBT/BCIP (Biochain) and counterstained with Nuclear Fast Red. The intensity of miR-193a-5p expression was quantified by HistoQuest Cell Analysis software.

### Immunohistochemistry

Tissue sections were dewaxed and rehydrated. Antigen retrieval was performed by heating the sections in 0.01 M sodium citrate buffer (pH 6.4). Endogenous peroxidase activity was blocked by immersion in 3% H_2_O_2_/methanol. Then, the sections were blocked in 5% normal goat serum/1 × PBS. The sections were incubated with an ERBB2 antibody (Abcam, ab-16901) followed by a biotinylated secondary antibody. The signals were revealed using the standard avidin-biotin-peroxidase complex method (ABC Elite) according to the manufacturer's instructions. Immunoreaction products were visualized using 3,3′-diaminobenzidine substrate (DAB, Sigma). The intensity of protein expression was quantified and analyzed by HistoQuest Cell Analysis software.

### CCRT-resistant cell generation

KYSE70 and CE48T CCRT-resistant cells were generated through a stepwise increase in cisplatin and irradiation. Cells treated with 1 μM cisplatin for 3 days were passaged through cisplatin-free medium. Upon reaching confluence, the cells were treated with a higher concentration of cisplatin. The dose of cisplatin was gradually increased with every few passages over a period of 2 months until cisplatin reached a concentration of 35 μM. During this period, the cells were irradiated with 6 MV photons. A total of 35 Gy was given in 7 fractions of 5 Gy each.

### MiRNA array hybridization and analysis

Total RNAs were isolated from ESCC and adjacent normal tissue by TRIzol reagent (Invitrogen). The isolated RNAs were labeled with dye at the 3′ position using the nCode Rapid miRNA Labeling System (Invitrogen). MiRNA arrays were generated on glass slides using nCode Human miRNA Microarray V3 (Invitrogen). The 710 mature miRNA complementary oligonucleotides were assembled and integrated into our microarray. The tagged RNAs were hybridized to the microarray slides at 52°C for 16 hr. After hybridization, the miRNA arrays were scanned and analyzed using a GenePix 4000A array scanner (Axon Instruments). All data were normalized to control miRNA. The quantification data are shown as the ratio of tumor to normal fluorescence intensity for each miRNA.

### Construction of the 3′UTR luciferase plasmid and reporter assays

The wild type ERBB2 3′UTR was amplified from cDNA with the primers ERBB2 3′UTR SpeI (5′- GA CAACTAGTACCAGAAGGCCAAGTCCGCA-3′) and ERBB2 3′UTR HindIII (5′-GACAAAGCTTAGCTG TTTTCCAAAATATAT-3′). The PCR products were then cloned into the pMIR-REPORT luciferase vector (Ambion). Mutant ERBB2-3′UTR was constructed by two-step site directed mutagenesis with the primers ERBB2 3′UTR mutant F (5′-AAATAAAGAGGGAGGGGGA GAATGGGTGTT-3′) and ERBB2 3′UTR mutant R (5′- CTCCCCCTCCCTCTTTATTTCATCTTTAAA-3′). All constructs were sequenced before further analysis. For the reporter assay, cells were transiently transfected with the wild type or mutant reporter plasmids. At 48 hours after transfection, the reporter assay was performed using the Dual-Luciferase Reporter Assay system (Promega).

### Herceptin treatment

KYSE70 cells were treated with Herceptin. Cells were incubated in serum-free medium for 60 minutes before Herceptin treatment, with several concentrations. At 72 hours after treatment, the MTT assay was used to measure cell viability. To investigate the effect of Herceptin combined with radiation treatment, cells were incubated in 300 μg/ml Herceptin/ 3% FBS RPMI-1640 medium. At 72 hours after treatment, the foci formation assay was used to measure radiosensitivity.

### Statistical analysis

All observations were confirmed by at least three independent experiments. The results were presented as the mean ± SE. All statistical analyses were performed using SPSS software (SPSS Inc.). We used a two-tailed, paired Student's *t*-test for all pair-wise comparisons. The correlation of the intensity of miR-193a-5p and ERBB2 was determined using Spearman's test. The survival curves were analyzed with the Kaplan-Meier method and compared with a log-rank test. The comparison of intensity of miR-193a-5p in normal and tumor tissues was determined using a Mann-Whitney test. The cell viability of Herceptin treatment groups was compared with two-way ANOVA.

## SUPPLEMENTARY MATERIALS FIGURES


