# Supplementary material for: MiR-193a-5p/ERBB2 act as concurrent chemoradiation therapy response indicator of esophageal squamous cell carcinoma
Source: Oncotarget. 2016 May 18;7(26):39680–93. doi: 10.18632/oncotarget.9444 (PMC5129962; doi:10.18632/oncotarget.9444)
Supplement: Supplementary file 1 [file oncotarget-07-39680-s001.pdf]

MiR-193a-5p/ERBB2 act as concurrent chemoradiation therapy response indicator of esophageal squamous cell carcinoma

Supplementary Materials

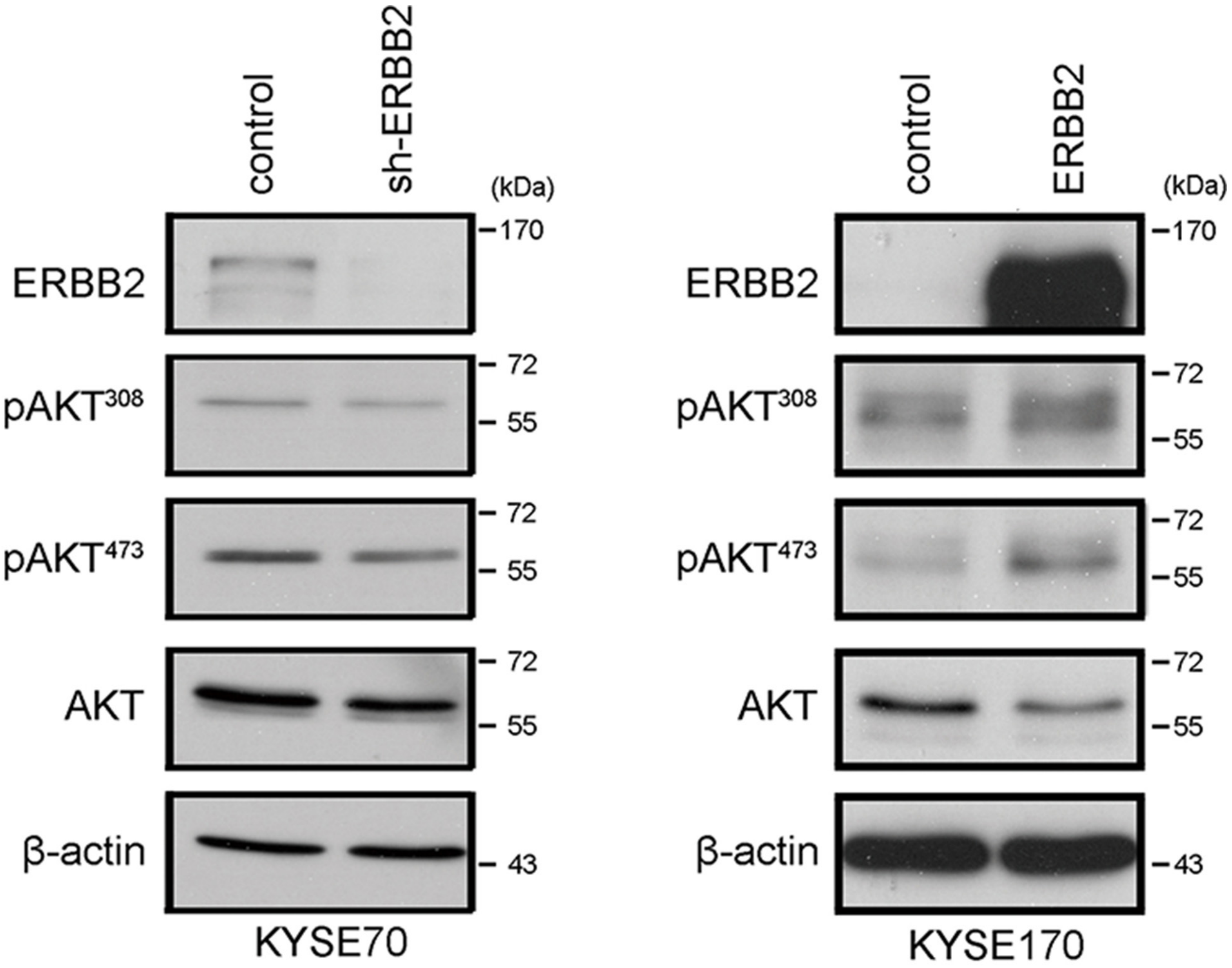

Supplementary Figure S1: The ERBB2 downstream AKT pathway is altered in ERBB2-down-regulated and ERBB2-overexpressing KYSE cells. Western blotting showed the expression of ERBB2, $pAKT^{308}$, $pAKT^{473}$, AKT, and β-actin in ERBB2-down-regulated and overexpressing KYSE70 and KYSE170 cells, respectively.

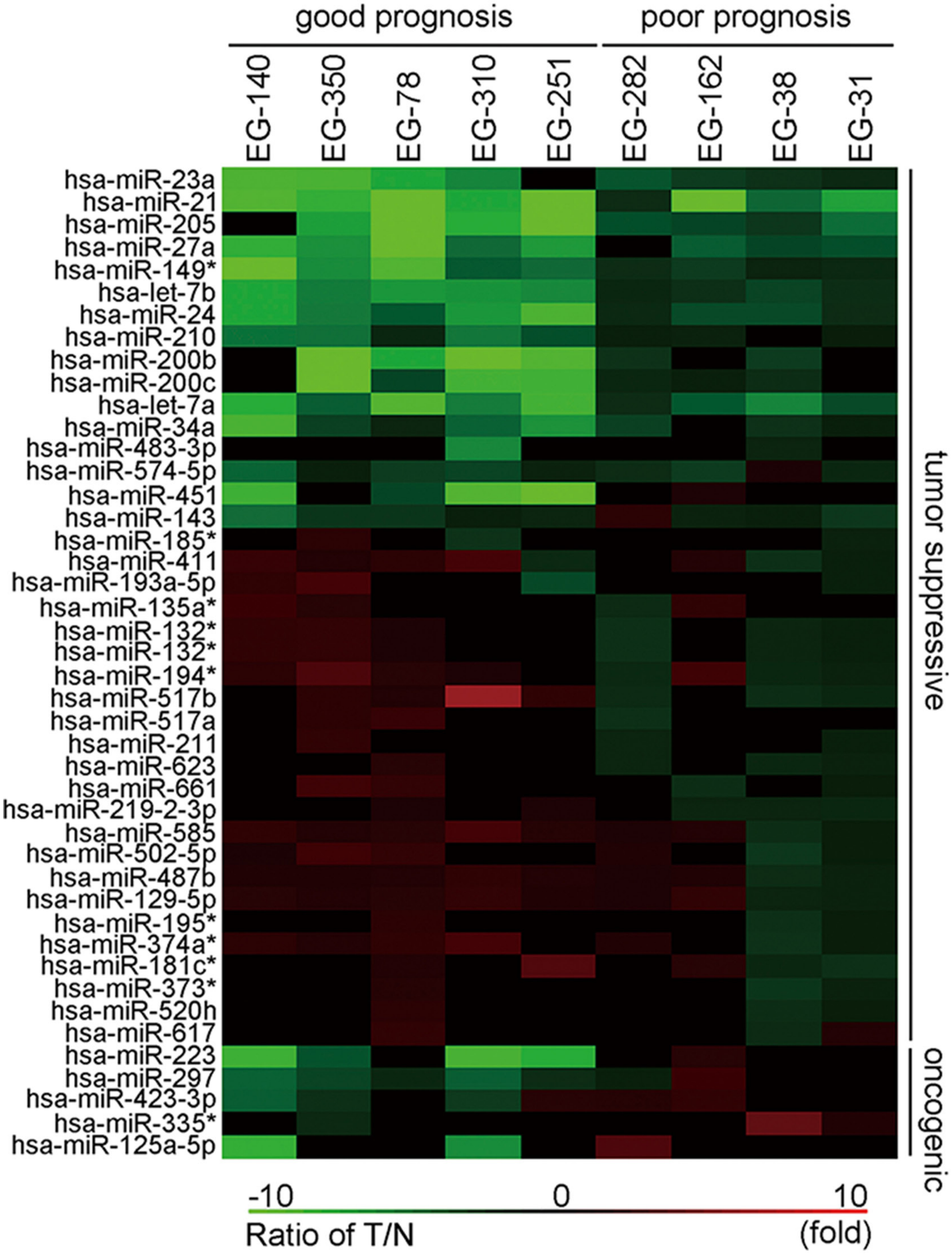

Supplementary Figure S2: Cluster analysis of miRNAs in a human microRNA array comparing nine paired ESCC samples. The heat map shows differential expression levels of 39 tumor-suppressor miRNAs and 5 oncogenic miRNAs. Red indicates higher miRNA expression in the tumor portion; green indicates lower miRNA expression in the tumor portion.

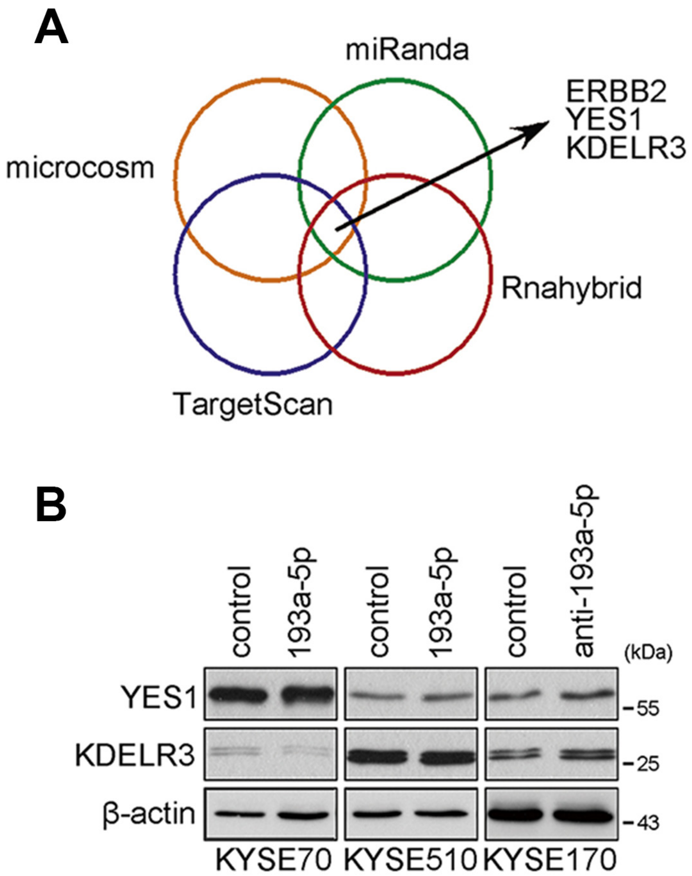

Supplementary Figure S3: ERBB2, but not YES1 and KDELR3, is inhibited by miR-193a-5p in KYSE cells. (**A**) Bioinformatics websites were used to predict the target genes of miR-193a-5p. ERBB2, YES1, and KDELR3 were three potential genes. (**B**) Western blotting showed the expression of YES1 and KDELR3 in KYSE cells. The expression of YES1 was similar after miR-193a-5p manipulation in KYSE cells. The expression of KDELR3 in miR-193a-5p-overexpressin KYSE70 was slightly decreased; however, the expression of KDELR3 in KYSE510 and KYSE170 was similar after miR-193a-5p manipulation. β-actin was used as an internal control.

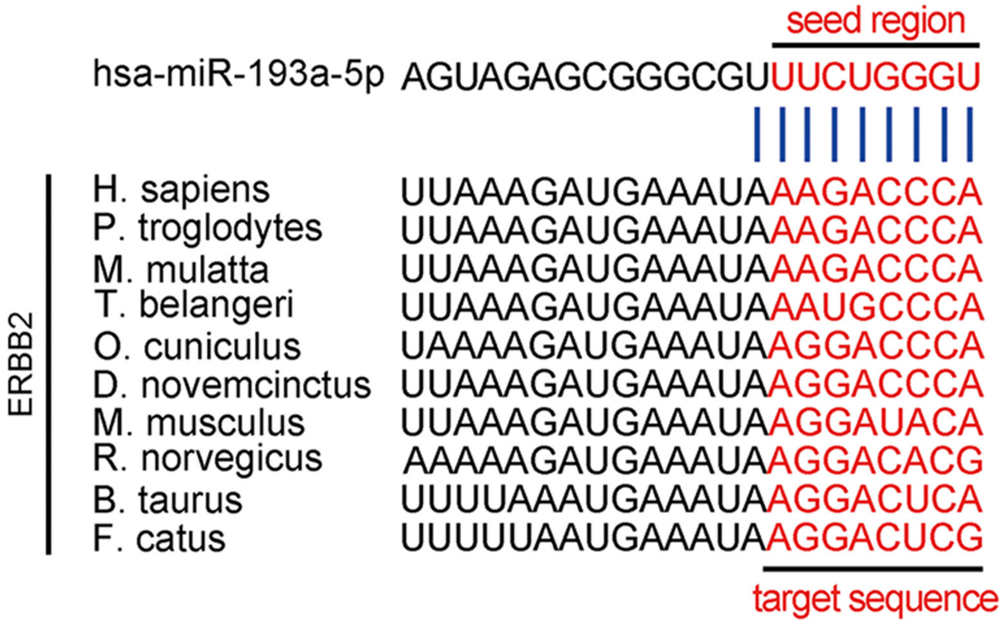

Supplementary Figure S4: The ERBB2 target sequence in the 3'UTR is highly conserved among different mammals. The seed region of miR-193a-5p was perfectly complementary with human and nine other mammalian ERBB2 3′UTRs.

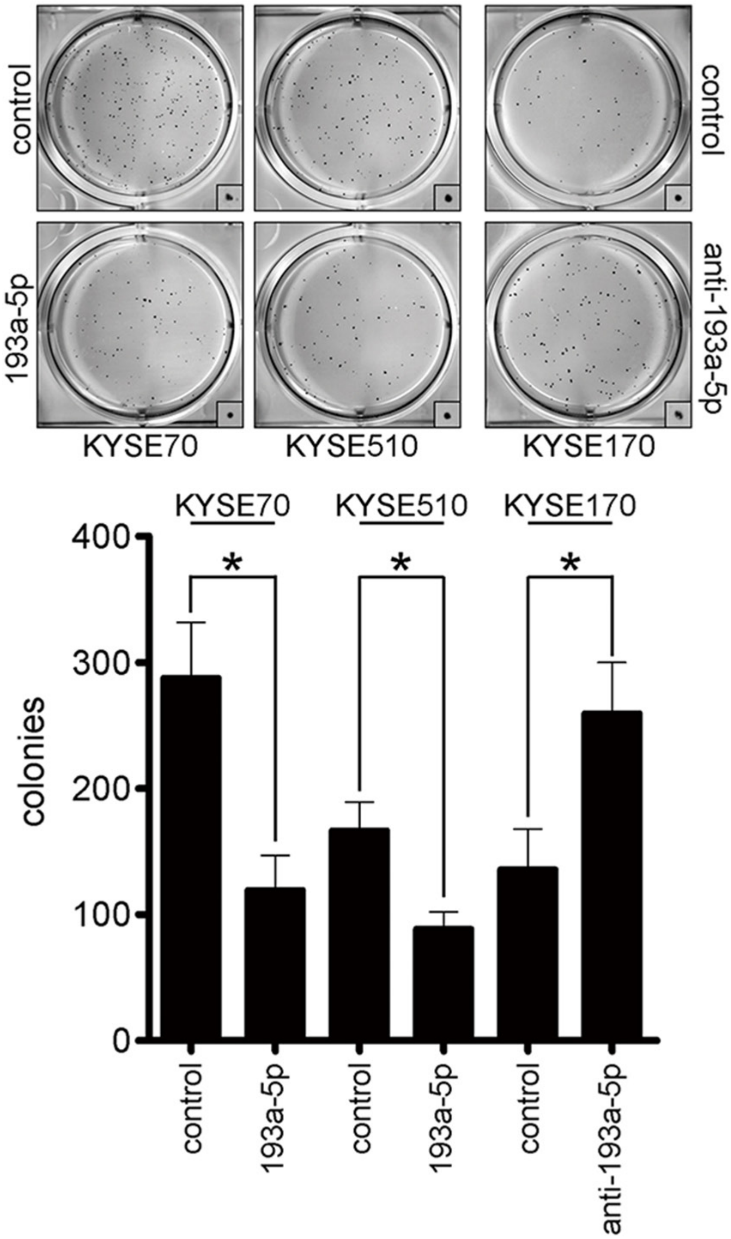

Supplementary Figure S5: MiR-193a-5p up-regulation suppresses anchorage-independent tumor growth in ESCC. The effect of miR-193a-5p on *in vitro* anchorage-independent tumor growth was analyzed using a soft agar assay in KYSE cells. MiR-193a-5p up-regulation significantly suppressed colony formation in KYSE70 and KYSE510 cells. MiR-193a-5p down-regulation significantly enhanced colony formation in KYSE170 cells (*$P < 0.05$).

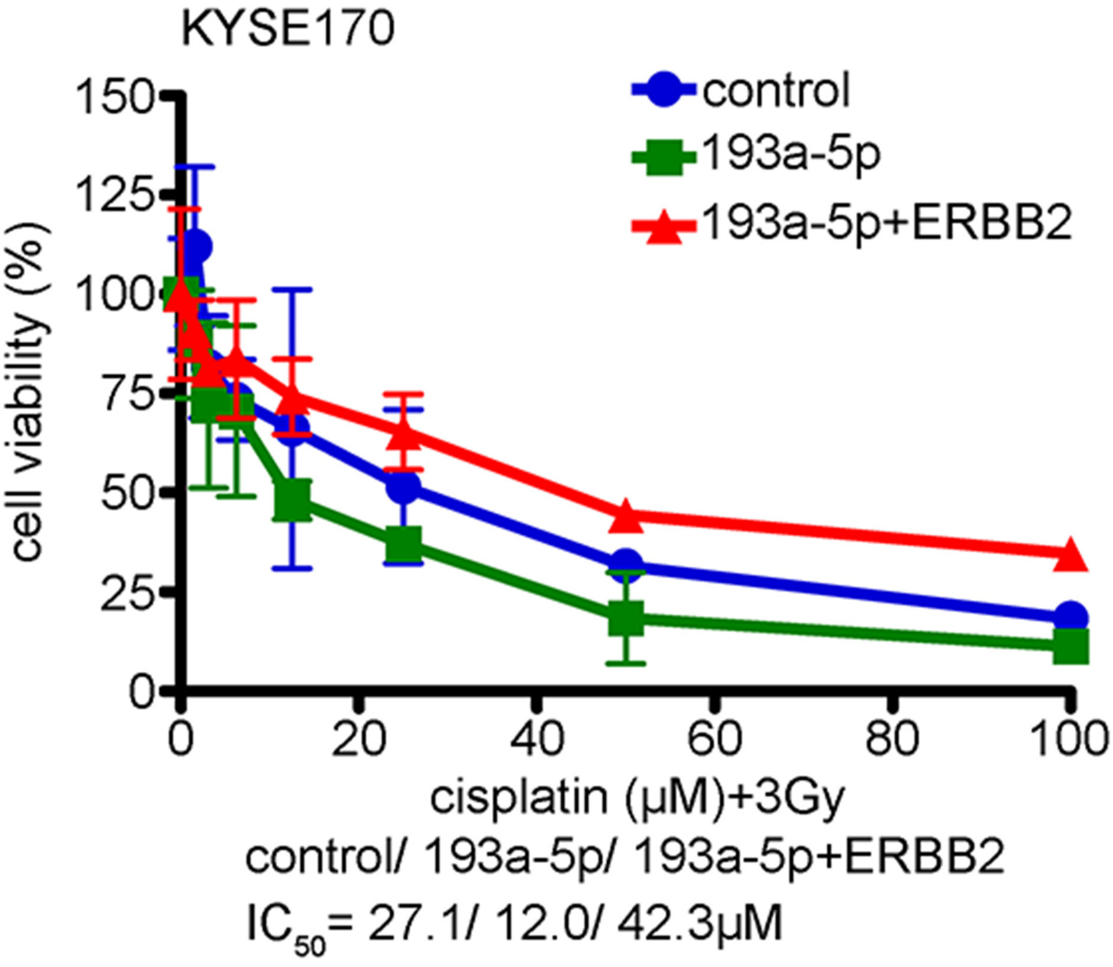

Supplementary Figure S6: MiR-193a-5p enhances CCRT sensitivity through ERBB2 dependent signaling. ERBB2 overexpressing and parental KYSE170 cells were transiently transfected with miR-193a-5p precursors. The MTT assay was used to evaluate CCRT sensitivity after treatment.

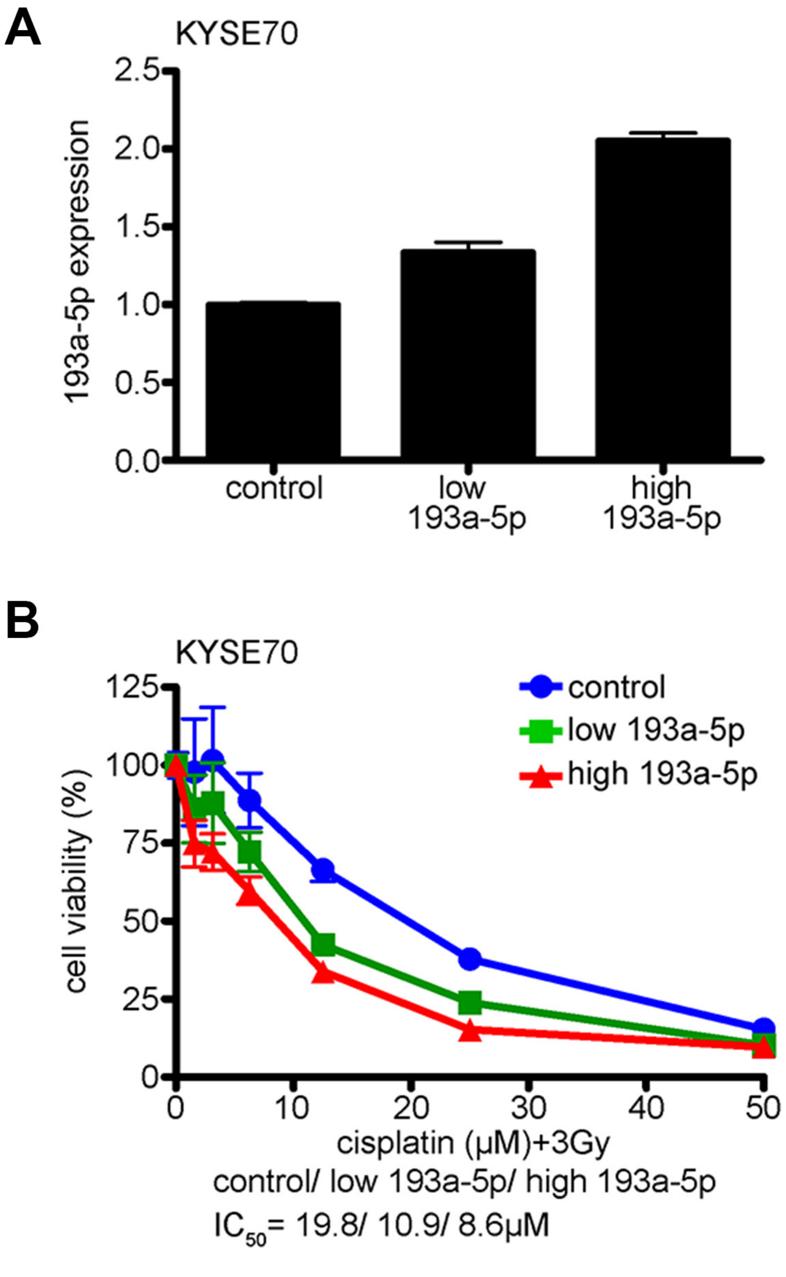

Supplementary Figure S7: MiR-193a-5p enhances CCRT sensitivity in a dose dependent manner in KYSE70 cells. (**A**) Real-time PCR showed the expression of miR-193a-5p in KYSE70 cells. (**B**) The MTT assay was used to analyze CCRT responses in KYSE70 cells with low and high miR-193a-5p expression.

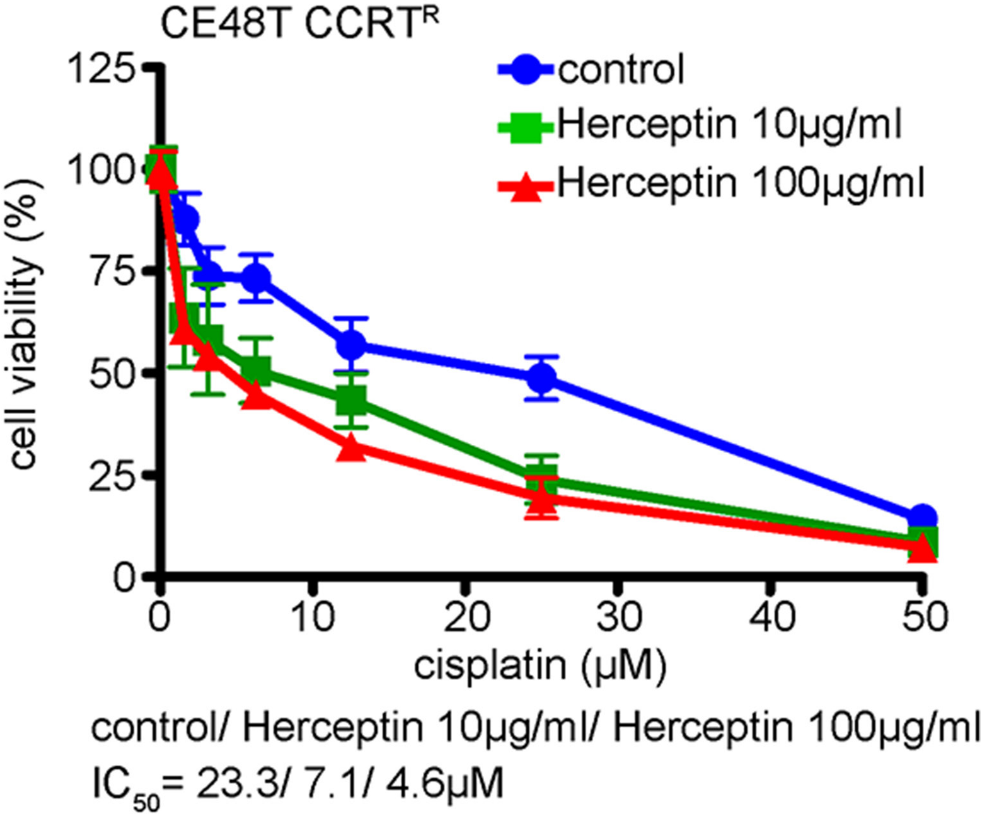

Supplementary Figure S8: Herceptin treatment enhances cisplatin sensitivity in CCRT resistant cells. CCRT resistant cells were treated with Herceptin (10 and 100 µg/ml). The MTT assay was used to measure cisplatin sensitivity after treatment.

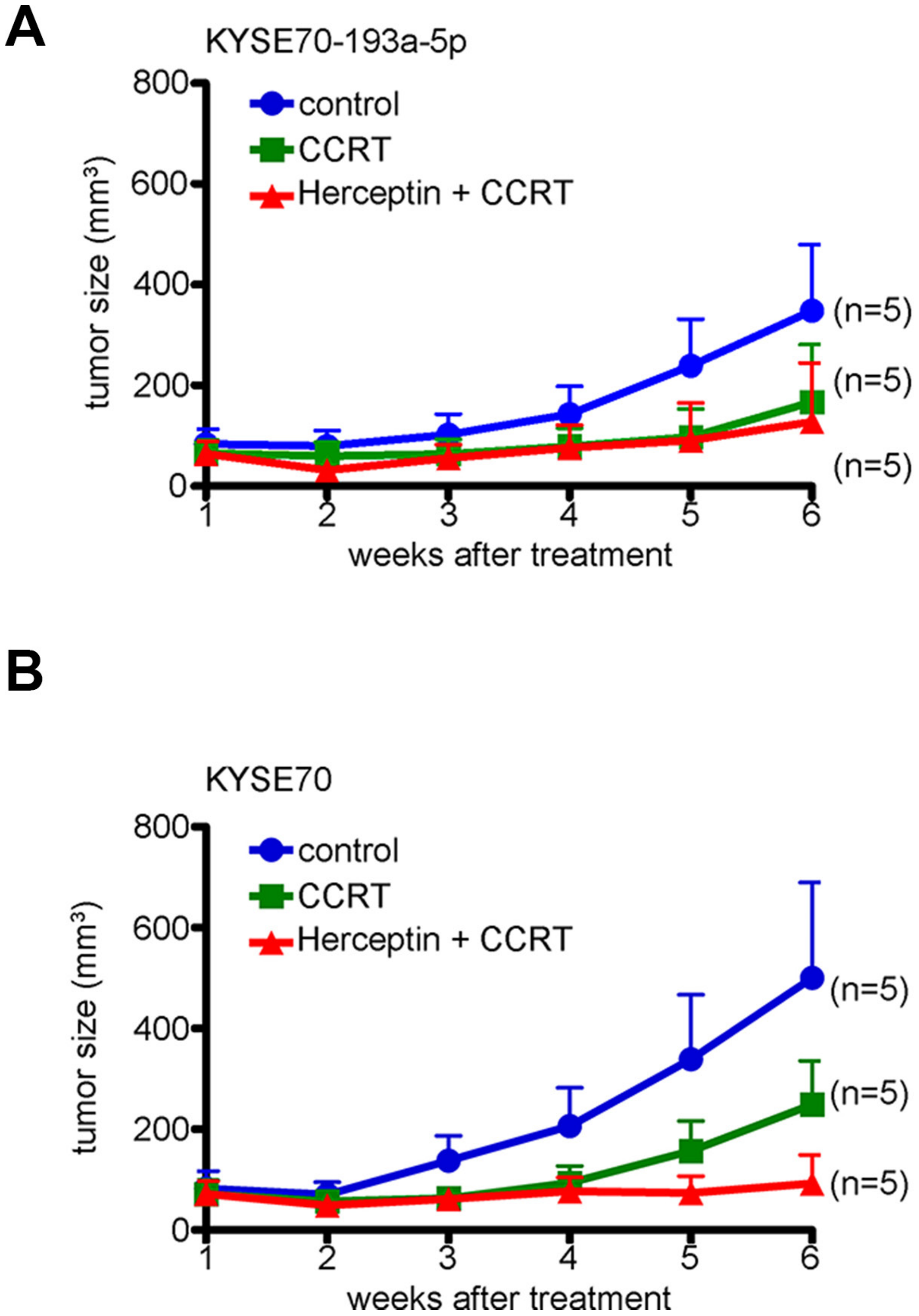

Supplementary Figure S9: Herceptin treatment enhances CCRT sensitivity *in vivo*. A xenograft mouse model was used to analyze the effect of Herceptin on CCRT sensitivity: (**A**) MiR-193a-5p overexpressing; (**B**) parental KYSE70 cells.

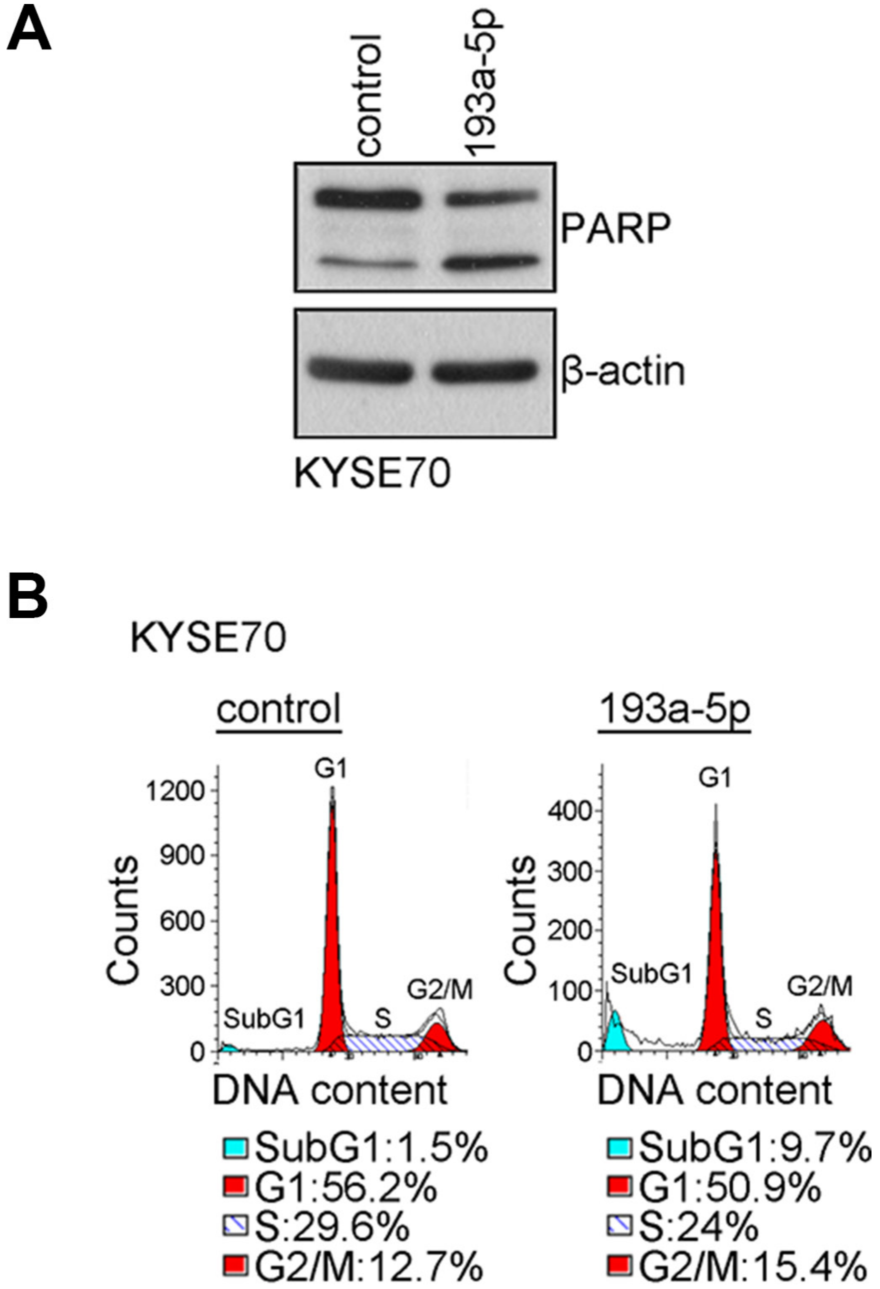

Supplementary Figure S10: MiR-193a-5p overexpression enhances apoptosis in KYSE70 cells. (**A**) Western blotting was used to detect the expression of PARP in KYSE70 parental and miR-193a-5p overexpressed cells. The expression of β-actin is shown as an internal control. (**B**) Flow cytometry was used to analyze the stages of cell cycle. Parental and miR-193a-5p overexpressing KYSE70 cells were stained with PI. Cell cycle stages were determined according to DNA content. The SubG1 population represents apoptotic cells.
